# Supplementary material for: The Cysteine-Rich Peptide Snakin-2 Negatively Regulates Tubers Sprouting through Modulating Lignin Biosynthesis and H2O2 Accumulation in Potato
Source: Int J Mol Sci. 2021 Feb 25;22(5):2287. doi: 10.3390/ijms22052287 (PMC7956376; doi:10.3390/ijms22052287)
Supplement: Supplementary file 1 [file ijms-22-02287-s001.zip › ijms-1091865-supplementary/Supplementary Figures.doc]

**Supplementary Figures**

Figure S1. Sequence analysis of StSN2. The coding sequence was identified by amplification of cDNA and gDNA in wild-type (WT, *Chuanyu*10). The corresponding protein sequence was translated at http://expasy.org/tools/dna.html.

Figure S2. CoIP analysis. All candidates proteins were coimmunoprecipitated by the anti-SN2 antibody in RNAi line 7 and OE line 27.

Figure S3. Analysis of *StSN2* expression in different tissues, storage periods and hormone treatments. (A) *StSN2* transcription level was detected in root, stem, leaf and tuber. (B) StSN2	accumulation was analysed by western blot in root, stem, leaf and tuber. (C) *StSN2* expression was investigated by qPCR assay during the course of the storage process. (D) The expression of *StSN2* was induced or repressed under ABA, GA_3_ and BR. (E) The expression alteration of *ABI3* and *ABI5* in *StSN2* transgenic potato lines. Data are means ± SD of three biological replicates. Different lowercase letters indicate significant difference at *P* < 0.05 by Duncan’s test analysis.

Figure S4. Relative expression of *rbohA* and *rbohB*. The effect of *StSN2* on *rbohA* and *rbohB*, the key genes for ROS synthesis in potato, was investigated in RNAi lines, OE lines and wild-type (WT) tuber.
